# Autoimmune Hepatitis After Successful Treatment of Chronic Hepatitis C Virus Infection with Direct-Acting Antivirals: A Pediatric Case Report

**DOI:** 10.3390/pathogens14121244

**Published:** 2025-12-05

**Authors:** Ewa Talarek, Małgorzata Aniszewska, Anna Dobrzeniecka, Jakub Kmiotek, Maria Pokorska-Śpiewak

**Affiliations:** 1 Department of Children’s Infectious Diseases, Medical University of Warsaw, Wolska 37, 01-201 Warsaw, Poland; malgorzata.aniszewska@wum.edu.pl (M.A.); anna.dobrzeniecka@wum.edu.pl (A.D.); maria.pokorska-spiewak@wum.edu.pl (M.P.-Ś.); 2 Department of Pediatric Infectious Diseases, Regional Hospital of Infectious Diseases in Warsaw, Wolska 37, 01-201 Warsaw, Poland; 3 Department of Gastroenterology, Hepatology, Nutritional Disorders and Pediatrics, Children’s Memorial Health Institute, Al. Dzieci Polskich 20, 04-730 Warsaw, Poland; j.kmiotek@ipczd.pl

**Keywords:** hepatitis C virus, autoimmune hepatitis, direct-acting antivirals

## Abstract

More than 3 million children are infected with hepatitis C virus (HCV) worldwide. Therapies with direct-acting antivirals (DAAs) are characterized by high efficiency and acceptable tolerability. Rare cases of autoimmune hepatitis (AIH) following HCV elimination have been reported in adults. Here, we present the first pediatric case of AIH after successful treatment with DAAs. A girl, born in 2012, was diagnosed with vertical HCV infection in 2013. In 2023, she was treated with the DAA glecaprevir/pibrentasvir. HCV RNA was undetectable after 4 weeks of treatment and at the end of treatment (EOT). However, at the EOT, the aminotransferase concentration elevated with further increase, despite a confirmed sustained viral response (SVR) 12 weeks after the EOT. Gamma-globulins were elevated, with positive anti-nuclear antibodies (ANA) and anti-liver kidney microsome (LKM) antibodies. Other causes were excluded. Elastography revealed no fibrosis. Aminotransferase levels decreased but did not normalize. A liver biopsy was performed, confirming a diagnosis of AIH. Immunosuppressive therapy with prednisone and azathioprine resulted in normalization of aminotransferase levels, and the titers of both ANA and LKM antibodies decreased. Monitoring aminotransferase levels should not be omitted in patients after successful DAA treatment of HCV infection.

## 1. Introduction

More than 3 million children are infected with hepatitis C virus (HCV) worldwide [1]. Chronic HCV infection may result in liver inflammation and fibrosis, increasing the risk of cirrhosis and hepatocellular cancer. HCV is also recognized as a trigger of autoimmune processes in some patients. It can manifest as autoimmune hepatitis (AIH), as extrahepatic disorders associated with chronic hepatitis C (CHC), or only in the presence of anti-smooth muscle antibodies (ASMA) and anti-nuclear antibodies (ANA) with no clinical features [2].

However, changes in the immune system may be caused not only by HCV infection but also by the recovery of innate and adaptive immune responses following elimination of HCV [3,4]. Reports of AIH cases after HCV RNA clearance achieved by treatment with direct-acting antivirals (DAAs) are rare [5,6,7,8,9,10]. DAA regimens, which are highly effective and safe, are currently the mainstay of treatment for both adults and children with CHC [11,12]. Here, we report the first pediatric case of AIH following the successful treatment of CHC with DAAs.

## 2. Case Report

**Figure 1:**
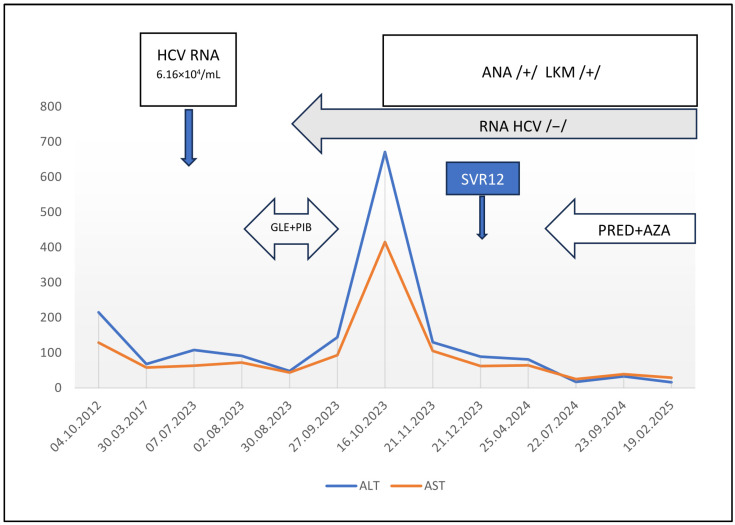
Clinical course of the patient. ALT—alanine aminotransferase; AST—aspartate aminotransferase; ANA—anti-nuclear antibodies; LKM—liver and kidney microsomal antibodies; /+/ positive; /−/ negative; GLE+PIB—treatment with glecaprevir and pibrentasvir; PRED+AZA—treatment with prednisone and azathioprine; SVR12—sustained virologic response 12 weeks after treatment.

A girl, born in 2012, was diagnosed with vertical HCV genotype 1b infection at the age of 7 months (in 2013). She was monitored, and her aminotransferase levels remained elevated, with alanine aminotransferase (ALT) concentrations ranging from 181–219 U/L and aspartate aminotransferase (AST) concentrations ranging from 79–129 U/L. In 2017, ANA were detected at a low titer of 1:320, during routine autoantibody examination in children with CHC, including ANA, ASMA and anti-liver kidney microsome (LKM) antibodies. At that time, ALT and AST levels were only slightly elevated at 68 U/L and 58 U/L, respectively. In 2023, the girl was eligible for 8 weeks of DAA treatment with 250 mg glecaprevir/100 mg pibrentasvir (GLE/PIB) daily. Before initiation of the treatment, ALT and AST levels were 91 U/L and 47 U/L, respectively, and the HCV RNA load was 6.16 × 10^4^/mL. Elastography revealed no fibrosis, with a liver stiffness measurement (LSM) of 5.4 kPa, which corresponded to F0/F1 on the Metavir scale [13]. After the first 4 weeks of treatment, ALT and AST levels decreased to 48 U/L and 44 U/L, respectively, and HCV RNA was not detected. At the end of treatment (EOT), aminotransferase levels were elevated (ALT: 144 U/L and AST: 93 U/L), and HCV RNA remained negative. One month later, ALT and AST levels increased to 671 U/L and 415 U/L, respectively. The bilirubin level and prothrombin time were normal. The patient was in good general condition and reported no complaints, and physical examination revealed no abnormalities. No other causes of hepatitis (e.g., infection with hepatitis A virus, hepatitis B virus, Epstein–Barr virus, cytomegaly virus, herpes simplex virus, adenovirus; or use of drugs (other than DAAs) and herbal remedies were identified. The proteinogram revealed increased levels of gamma-globulins (14.27 g/L; normal range 6.0–12.7 g/L) with positive ANA and anti-liver kidney microsome (LKM) antibodies at titers of 1:1280 and 1:2560, respectively; ASMA were negative. AIH was suspected; however, the aminotransferase levels decreased, and the liver biopsy was postponed. A sustained viral response (SVR) was confirmed 12 weeks after the EOT, aminotransferase levels were only slightly elevated (ALT 89 U/L and AST 62 U/L), the gamma-globulin level was normal, but the LKM titer increased to 1:5120; the ANA titer remained stable at 1:1280, ASMA were still negative. Wilson disease and alpha1-antitrypsin deficiency were excluded. The patient was carefully followed up. Although no clinical symptoms appeared, abnormal aminotransferase levels were still detected, and a liver biopsy was performed in 2024. Histopathologic examination revealed interface hepatitis with inflammatory infiltrates composed of lymphocytes and plasma cells in widened porto-biliary spaces, segmentally crossing the limiting plate and invading the parenchyma, and fibrosis with the formation of segmental porto-portal bridges. The findings were suggestive of AIH, and immunosuppressive treatment with 50 mg prednisone daily and 50 mg azathioprine daily was started (the body weight was 45 kg). This treatment resulted in the normalization of aminotransferase levels; the gamma-globulin level remained normal, and both the LKM and ANA titers decreased to 1:640 (Figure 1). The improvement in immunosuppressive treatment confirmed the diagnosis of AIH. The patient is still on maintenance treatment with 10 mg prednisone every 48 h and 25 mg azathioprine daily, with monitoring complete blood count and 6-thioguanine nucleotide concentrations in erythrocytes. No adverse effects of treatment have been observed. Elastography reveals no fibrosis (with LSM of 4.2 kPa).

## 3. Discussion

The development of autoimmune processes results from the interplay between genetic (such as HLA-DR3, HLA-DR-4 or HLA-DR7) and environmental factors, such as viral infections or drug use [14]. Chronic immune stimulation by HCV may cause autoimmunity with the development of several autoantibodies, although in some patients, it does not result in clinically overt autoimmune disease, e.g., AIH [2]. In the past, the risk of exacerbation of autoimmune processes caused by the use of interferon in the treatment of CHC was a reason to defer therapy. Currently, patients with CHC are both effectively and safely treated with DAAs. Some reports have provided evidence that elimination of HCV can result in the clearance of autoantibodies and the improvement of coexisting autoimmune diseases [15,16]. On the other hand, there are rare cases of AIH during or after HCV elimination in adult patients with or without previous autoimmunity [5,6,7,8,9,10]. The characteristics of those patients and our patient are presented in Table 1.

Most of the reported patients, including ours (8 of 9), were female. Similarly, most of them (5 of 6 in whom data are provided) were infected with the HCV genotype 1/1b, and this genotype is the most common in Italy, Spain, Japan and Poland [17], i.e., the countries where the case reports come from. The identified patients received various DAAs. Most patients were treated with sofosbuvir-based regimens, but our patient underwent therapy with GLE/PIB. The duration of AIH onset in relation to treatment initiation varied from 2 weeks to 5 years (in two cases it was not reported). Four patients were previously diagnosed with an autoimmune disease (e.g., idiopathic thrombocytopenic purpura, autoimmune hypothyroidism, autoimmune hemolytic anemia). Autoantibodies were detected before DAA treatment in three other patients (including our patient). In all patients, ANA were present, and in some cases they were accompanied by other antibodies typically found in patients with type I AIH. Our patient was the only patient with positive LKM antibodies, which are diagnostic for AIH type II in the absence of HCV infection [14,18]. In adult patients, the AIH diagnosis was supported by scores calculated according to the original and the simplified International Autoimmune Hepatitis Group (IAIHG) scoring systems [19,20]. The use of the simplified IAHG scale for diagnosing AIH in pediatric patients is limited [18] due to the different course of the disease compared to adults. Due to the patient’s positive ANA antibody titer (1:640), histopathological findings consistent with a diagnosis of AIH, and the exclusion of active viral infections, she was assigned a score of 6 points (which, in interpretation, indicates a probable diagnosis of AIH). The detection of other tissue antibodies (LKM 1:640; LKM1 positive) and the response to treatment confirmed the diagnosis. However, it should be emphasized that in pediatric practice, the most important criterion for diagnosing AIH is the histopathological result. All reported patients were treated with immunosuppressive drugs. Our patient is treated with prednisone and azathioprine which is currently the standard and primary treatment for pediatric AIH is steroids and azathioprine. If the medications are well tolerated and effective (normalization of laboratory markers of hepatitis, favorable trends in liver elastography, and no changes in the diagnosis due to overlap syndromes), histopathological evaluation of treatment outcomes is performed after two years. Unlike in adult patients, mycophenolate mofetil (MMF) is not primarily used in children (either as monotherapy or in combination with a steroid). Its use is considered in cases of failure or intolerance to prior treatment. This is due to the currently undetermined efficacy of MMF in this age group. Additionally, it is not reimbursed for this condition in Poland. In 7 of the 9 patients, clinical improvement and normalization of ALT, AST and immunoglobulin IgG levels were achieved. In one patient primary sclerosing cholangitis developed 3 years later [9] and one died due to sepsis and multiorgan failure [10].

The reported patients with AIH following CHC treatment with DAAs constitute a heterogeneous group, and identifying the underlying mechanism is not possible. At least two potential factors may be taken into consideration. First, chronic HCV infection affects both congenital and adaptive immunity. Alterations include dysfunction and a reduced diversity of natural killer (NK) cells, an increased number of regulatory CD4+ T cells, and the deletion of HCV-specific CD4+ T cells and HCV-specific CD8+ T-cell exhaustion, all of which lead to the loss of effector function and increased expression of inhibitory markers. After rapid HCV clearance is achieved by DAA treatment, immune function is restored. Disruption of immune tolerance may cause autoimmune disease [3]. Additionally, mucosal-associated invariant T (MAIT) cells may participate in autoimmune processes. During chronic HCV infection, the number and function of peripheral and intrahepatic MAIT cells are depleted. After rapid elimination of HCV, the number of MAIT cells in the liver increases. The activation of MAIT cells may promote inflammation and fibrosis [21]. Second, autoimmune processes may be triggered by drugs, leading to drug-induced autoimmune liver disease (DIAILD), such as AIH with drug-induced liver injury (DILI), drug-induced AIH (DI-AIH) or immune-mediated DILI (IM-DILI). The clinical features of DILI may be similar to those of AIH, with the presence of autoantibodies and high levels of immunoglobulin IgG; therefore, distinguishing between the two is difficult, even on liver biopsy [14,22]. DI-AIH was suspected in one of the reported cases discussed above [5]. The patient developed liver injury two months after the initiation of DAA treatment with elbasvir and grazoprevir. In our patient, another DAA treatment was used (GLE/PIB), but the time of onset was similar, and DI-AIH could not be excluded.

## 4. Conclusions

Although cases of AIH following DAA treatment are rare and the underlying mechanism is difficult to identify, it is worth considering such a risk, regardless of a previous history of autoimmunity. In all reported patients, the first and in some cases the only sign of liver injury was an increase in aminotransferase levels. Since DAAs are usually well tolerated, there is a tendency to reduce visits and tests during and after treatment. We suggest that monitoring aminotransferase levels should not be omitted in patients who have been successfully treated.

## Figures and Tables

**Table 1:** Characteristics of patients with AIH following DAA treatment—review of the literature.

Ref.	Age/ Sex	Previous Autoimmune Disease or Autoantibodies Positive	HCV Geno-Type	DAA	SVR	Time Between Start of DAA and Onset of AIH	Autoantibodies Positive
Matsumoto et al. [5]	81/F	no	1	EBR+GZR	yes	2 months	ANA 1:160
Covini et al. [6]	72/F	ITP	1b	SOF+LDV	yes	2 weeks	ANA 1:320; p-ANCA > 1:320
Cacciato et al. [7]	66/M	ANA > 1:320; ASMA 1:160	2	SOF+VLP	yes	one year	N/A
Monton et al. [8]	72/F	ANA+	1b	SOF+LDV	yes	3 years	ANA 1:1280; ASMA 1:640; anti-actin 1:640
Morihisa et al. [9]	74/F	no	1b	SOF+LDV	yes	6 months	ANA 1:80; ASMA 1:640
Malakar et al. [10]	57/F 46/F 67/F	autoimmune hypothyroidism autoimmune hypothyroidism autoimmune hemolytic anemia	N/A N/A N/A	SOF+DCV SOF+DCV SOF+DCV	yes yes yes	N/A N/A 5 years	ANA 1:80; ASMA 1:100 ANA 1:80; ASMA 1:80 ANA 1:80
Our case	12/F	ANA 1:100	1b	GLE-PIB	yes	2 months	ANA 1:1280; LKM 1:5120

AIH—autoimmune hepatitis; ANA—anti-nuclear antibodies; ASMA—anti-smooth muscle antibodies; DAA—direct-acting antivirals; DCV—daclatasvir; EBR—elbasvir; F—female; GLE—glecaprevir; GZR—grazoprevir; HCV—hepatitis C virus; ITP—idiopathic thrombocytopenic purpura; LDV—ledipasvir; LKM—anti-liver kidney microsome antibodies; M—male; N/A—not available; p-ANCA—peripheral anti-neutrophil cytoplasmic antibodies; PIB—pibrentasvir; SOF—sofosbuvir; SVR—sustained viral response; VLP—velpatasvir.

## Data Availability

Data available on a reasonable request due to ethical reasons. Patient’s records are kept in Department of Pediatric Infectious Diseases, Regional Hospital of Infectious Diseases in Warsaw, Poland. Further inquiries can be directed to the corresponding author.

## Abbreviations

The following abbreviations are used in this manuscript:

HCV	Hepatitis C virus
DAA	Direct-acting antiviral
AIH	Autoimmune hepatitis
EOT	End of treatment
SVR	Sustained viral response
ANA	Anti-nuclear antibodies
LKM	Liver kidney microsomal
CHC	Chronic hepatitis C
ASMA	Anti-smooth muscle antibodies
ALT	Alanine aminotransferase
AST	Aspartate aminotransferase
GLE	Glecaprevir
PIB	Pibrentasvir
LSM	Liver stiffness measurement
PRED	Prednizone
AZA	Azathioprine
ELB	Elbasvir
F	Female
GZR	Grazoprevir
ITP	Idiopathic thrombocytopenic purpura
LDV	Ledipasvir
DCV	Daclatasvir
M	Male
N/A	Not available
SOF	Sofosbuvir
VLP	Velpatasvir
IAIHG	International Autoimmune Hepatitis Group
NK	Natural killers
MAIT	Mucosal-associated invariant T
DIAILD	Drug-induced autoimmune liver disease
DILI	Drug-induced liver injury
DI-AIH	Drug-induced autoimmune hepatitis
IM-DILI	Immune-mediated drug-induced liver injury
MMF	Mycophenolate mofetil
